# Serial Changes of Serum Growth Factor Levels and Liver Regeneration after Partial Hepatectomy in Healthy Humans

**DOI:** 10.3390/ijms141020877

**Published:** 2013-10-17

**Authors:** Kazuyuki Matsumoto, Yasuhiro Miyake, Yuzo Umeda, Hiroshi Matsushita, Hiroaki Matsuda, Akinobu Takaki, Hiroshi Sadamori, Kazuhiro Nouso, Takahito Yagi, Toshiyoshi Fujiwara, Kazuhide Yamamoto

**Affiliations:** 1 Department of Gastroenterology and Hepatology, Okayama University Graduate School of Medicine, Dentistry, and Pharmaceutical Sciences, 2-5-1, Shikata-cho, Kita-ku, Okayama 700-8558, Japan; E-Mails: matsumotokazuyuki0227@yahoo.co.jp (K.M.); okahirosix@yahoo.co.jp (H.M.); akitaka@md.okayama-u.ac.jp (A.T.); nouso@cc.okayama-u.ac.jp (K.N.); kazuhide@md.okayama-u.ac.jp (K.Y.); 2 Department of Gastroenterological Surgery, Okayama University Graduate School of Medicine, Dentistry, and Pharmaceutical Sciences, 2-5-1, Shikata-cho, Kita-ku, Okayama 700-8558, Japan; E-Mails: y.umeda@d9.dion.ne.jp (Y.U.); hmat39@ybb.ne.jp (H.M.); sada@md.okayama-u.ac.jp (H.S.); 1957takahito@celery.ocn.ne.jp (T.Y.); toshi_f@md.okayama-u.ac.jp (T.F.)

**Keywords:** hepatectomy, hepatocyte growth factor, human, leptin, liver regeneration, macrophage colony-stimulating factor

## Abstract

This study aimed to investigate the associations of the serial changes of serum levels of various growth factors with liver regeneration after hepatectomy in healthy liver donors. Sixteen healthy liver donors who underwent conventional liver resection were included. Serum levels of various growth factors before hepatectomy and on postoperative day (POD) 1, 3, 5 and 7 were measured. Liver volume data calculated by multi-detector computed tomography using workstation. The ratio of remnant liver volume on POD 0 to liver volume before the operation was 51% ± 20%. The ratio of liver volume on POD 14 to liver volume on POD 0 were inversely correlated with remnant liver volume on POD 0 (*r* = −0.91). The ratio of liver volume on POD 14 to liver volume on POD 0 were significantly correlated with serum hepatocyte growth factor (HGF) levels on POD 1 (*r* = 0.54), serum leptin levels on POD 1 (*r* = 0.54), and serum macrophage colony-stimulating factor (M-CSF) levels on POD 5 (*r* = 0.76) and POD 7 (*r* = 0.80). These results suggest that early-phase elevation of serum levels of HGF, leptin and M-CSF may be associated with the acceleration of liver regeneration after hepatectomy in humans.

## 1. Introduction

Liver transplantation is the only curative treatment for end-stage liver diseases. However, in a setting of the shortage of liver grafts, many patients deteriorate as a result of disease progression or develop complications because of the lack of a timely suitable donor while waiting for a liver graft [1,2]. Thus, in addition to liver transplantation, new therapeutic agents for promoting liver regeneration are desired.

In animal models, the mechanisms of liver regeneration have been investigated in detail. Hepatocytes are primed by tumor necrosis factor (TNF)-α and interleukin (IL)-6 mainly produced by Kupffer cells, and then proliferation and cell growth of hepatocytes are induced in response to transforming growth factor-α, hepatocyte growth factor (HGF), and epidermal growth factor [3]. In addition, vascular endothelial growth factor (VEGF) and thrombopoietin (TPO) are shown to promote liver regeneration [4,5].

In humans, *in vivo* investigations of liver regeneration have been mainly performed in patients undergoing surgical resection of liver cancers or liver transplant recipients; however, underlying diseases and immunosuppressant after liver transplantation may influence liver regeneration. Until now, a few studies have shown that serum HGF and IL-6 levels are elevated on postoperative day (POD) 1 [6–8]. In individuals without the appropriate elevation of serum HGF levels after partial hepatectomy, postoperative liver failure develops more frequently [9]. Serial changes of serum VEGF and TPO levels after partial hepatectomy have been also investigated in healthy liver donors [7,10]. However, associations of these growth factors with liver regeneration have not been fully revealed.

Recently, because of the shortage of liver grafts from deceased donors, the number of living donor liver transplantation has increased. In living donor liver transplantation, healthy liver donors undergo typical and anatomical hepatectomy. So, the mechanisms of liver regeneration in healthy humans, which may be different from those in patients undergoing surgical resection of liver cancers, liver transplant recipients, and animal models, may be revealed. In this study, we investigated the serial changes of serum levels of various growth factors after partial hepatectomy and the associations of these changes of various growth factors with liver regeneration after the operation in healthy liver donors.

## 2. Results

### 2.1. Clinical Characteristics of Study Population

Clinical characteristics of 16 healthy liver donors are shown in Table 1. Preoperative liver function tests were within normal limit in all patients. Each donor did not require perioperative transfusion or suffer from any major operative complications after surgery.

Liver graft type and changes of liver volume before and after partial hepatectomy are summarized in Table 2. The ratio of remnant liver volume on POD 0 to liver volume before the operation was 51% ± 20%. The ratio of liver volume on POD 14 to liver volume before the operation was 76% ± 11%. Remnant liver volume per body weight on POD 0 were more in left graft donors than in right graft donors (15.6 ± 1.8 cm^3^/kg *versus* 7.7 ± 2.7 cm^3^/kg, *p* < 0.0001); however, the ratio of liver volume on POD 14 to liver volume on POD 0 was higher in right liver donors than in left liver donors (199% ± 42% *versus* 114% ± 8%, *p* = 0.0003). Ratio of liver volume on POD 14 to liver volume on POD 0 was inversely correlated with remnant liver volume on POD 0 (*r* = −0.91, *p* < 0.0001) and remnant liver volume per body weight on POD 0 (*r* = −0.95, *p* < 0.0001). On the other hand, the ratio of liver volume on POD 14 to liver volume on POD 0 was not associated with gender, age and body mass index.

### 2.2. Postoperative Changes of Laboratory Data and Liver Regeneration

**Figure 1:**
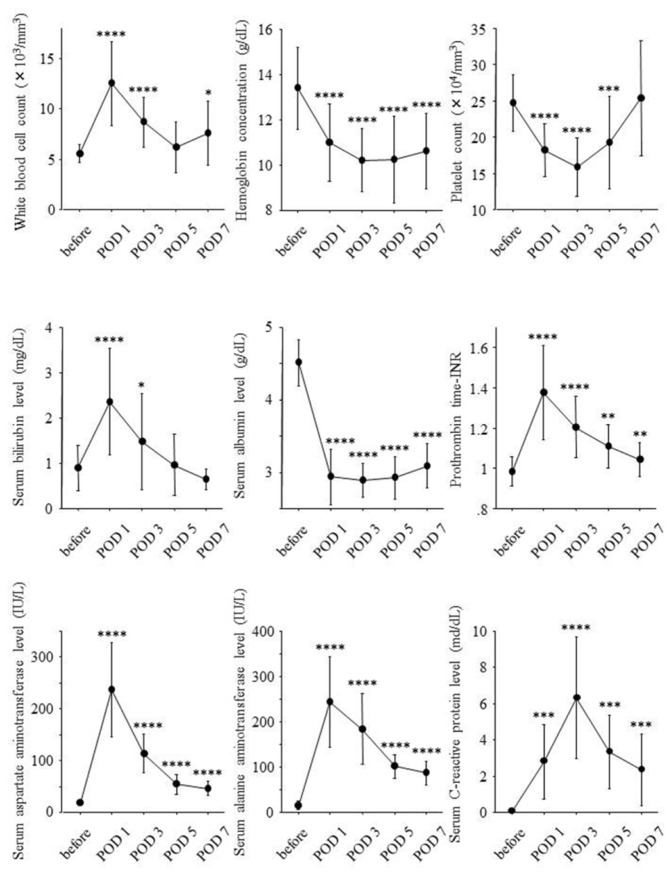
Serial changes of laboratory data during the clinical course. Laboratory data before hepatectomy and on postoperative day (POD) 1, 3, 5 and 7 were expressed as mean ± standard deviation. Before: before partial hepatectomy; POD: postoperative day; *: *p* < 0.05; **: *p* < 0.01; ***: *p* < 0.001; ****: *p* < 0.0001.

Serial changes of laboratory data before hepatectomy and on POD 1, 3, 5 and 7 are shown in Figure 1.

Liver resection rate was significantly correlated with white blood cell counts on POD 1 (*r* = 0.65, *p* = 0.005), serum bilirubin levels on POD 3 (*r* = 0.51, *p* = 0.045), serum albumin levels on POD 3 (*r* = 0.57, *p* = 0.020), serum aspartate aminotransferase levels on POD 3 (*r* = 0.63, *p* = 0.007) and POD 5 (*r* = 0.81, *p* = 0.0006), and prothrombin time-international normalized ratio (INR) on POD 3 (*r* = 0.71, *p* = 0.002) and POD 5 (*r* = 0.78, *p* = 0.004) but was inversely correlated with serum *C*-reactive protein levels (*r* = −0.67, *p* = 0.005). Remnant liver volume per body weight on POD 0 was inversely correlated with white blood cell counts on POD 1 (*r* = −0.61, *p* = 0.011), serum aspartate aminotransferase levels on POD 3 (*r* = −0.78, *p* = 0.0002) and POD 5 (*r* = −0.78, *p* = 0.019), and prothrombin time-INR on POD 3 (*r* = −0.68, *p* = 0.003) and POD 5 (*r* = −0.78, *p* = 0.003) but was significantly correlated with serum C-reactive protein levels (*r* = 0.66, *p* = 0.006).

**Figure 2:**
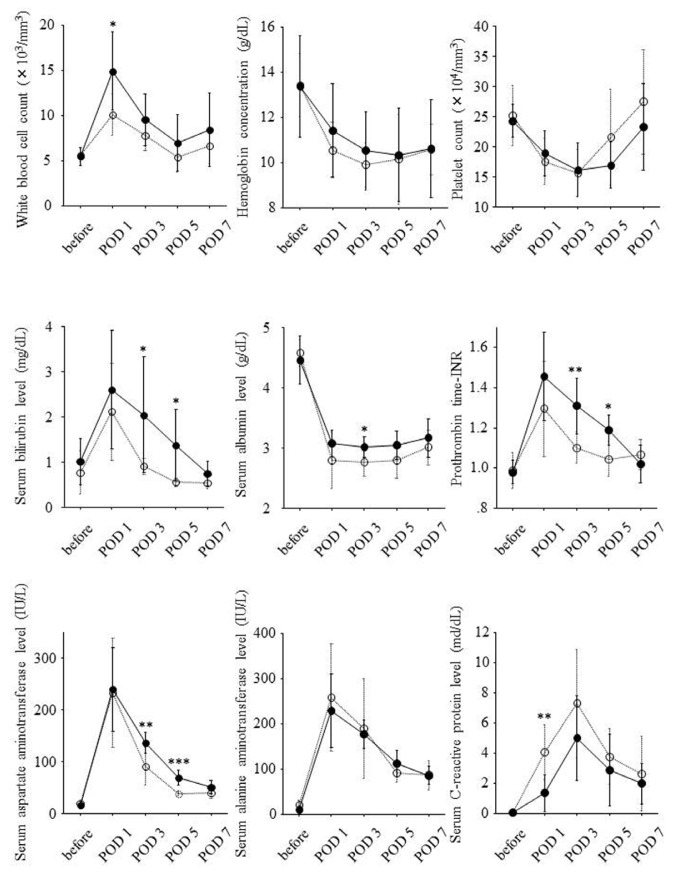
Associations of remnant liver volume per body weight on POD 0 with serial changes of laboratory data during the clinical course. Solid and dotted lines show serial changes of serum levels of each growth factor in eight patients with remnant liver volume per body weight on POD 0 of 10 cm^3^/kg or less and the other eight patients with remnant liver volume per body weight on POD 0 > 10 cm^3^/kg, respectively. Serum levels of each growth factor before hepatectomy and on POD 1, 3, 5 and 7 were expressed as mean ± standard deviation. Before: before partial hepatectomy; POD: postoperative day; *: *p* < 0.05; **: *p* < 0.01; ***: *p* < 0.001.

According to remnant liver volume per body weight on POD 0, 16 patients were divided into two groups. One group consisted of eight patients with remnant liver volume per body weight on POD 0 of 10 cm^3^/kg or less, and another group consisted of the other eight patients with remnant liver volume per body weight on POD 0 >10 cm^3^/kg. Serial changes of laboratory data in both the groups are shown in Figure 2. White blood cell counts on POD 1, serum bilirubin levels on POD 3 and 5, serum albumin levels on POD 3, serum aspartate aminotransferase levels on POD 3 and 5, and prothrombin time-INR on POD 3 and 5 were significantly higher in the eight patients with remnant liver volume per body weight on POD 0 of 10 cm^3^/kg or less. On the other hand, serum C-reactive protein levels on POD 1 were lower in this group.

Ratio of liver volume on POD 14 to liver volume on POD 0 was correlated with white blood cell counts on POD 1 (*r* = 0.63, *p* = 0.007), prothrombin time-INR on POD 3 (*r* = 0.62, *p* = 0.009) and POD 5 (*r* = 0.72, *p* = 0.010), and serum aspartate aminotransferase levels on POD 3 (*r* = 0.71, *p* = 0.002) and POD 5 (*r* = 0.67, *p* = 0.015). On the other hand, serum C-reactive protein levels on POD 1 were inversely correlated with ratio of liver volume on POD 14 to liver volume on POD 0 (*r* = −0.62, *p* = 0.012).

**Figure 3:**
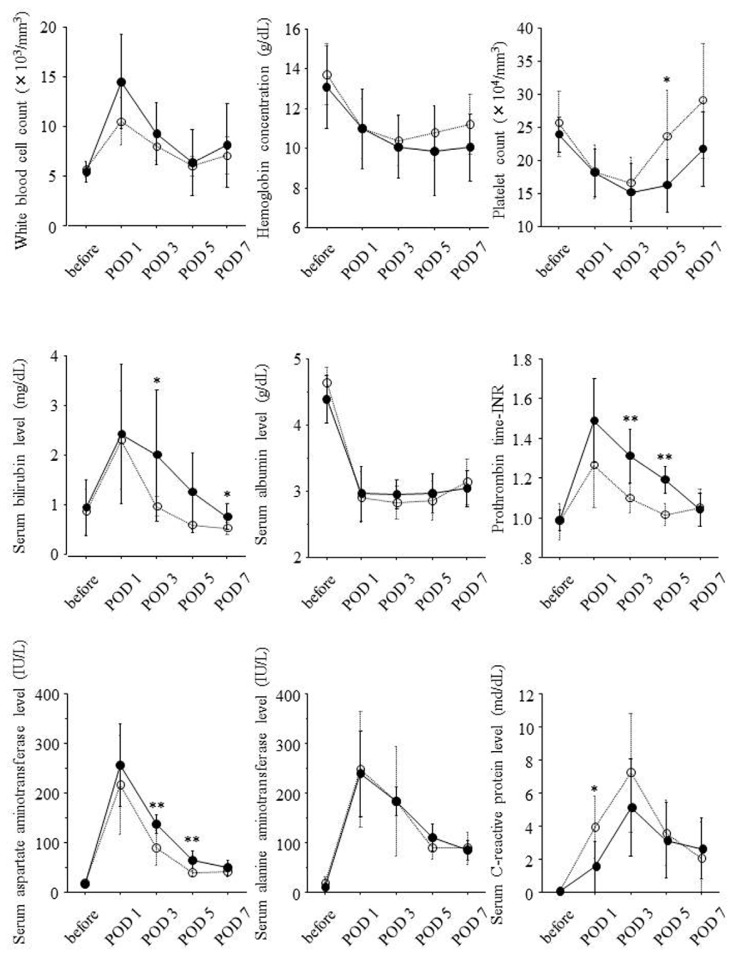
Associations of liver regeneration with serial changes of laboratory data during the clinical course. Solid and dotted lines show serial changes of laboratory data in eight patients showing ratio of liver volume on POD 14 to liver volume on POD 0 of 150% or higher and the other eight patients showing this ratio <150%, respectively. Serum levels of each laboratory data before hepatectomy and on POD 1, 3, 5 and 7 were expressed as mean ± standard deviation. Before: before partial hepatectomy; POD: postoperative day; *: *p* < 0.05; **: *p* < 0.01.

According to the ratio of liver volume on POD 14 to liver volume on POD 0, 16 patients were divided into two groups. Eight patients showing ratio of liver volume on POD 14 to liver volume on POD 0 of 150% or higher were classified into high liver regeneration group, and the others eight showing this ratio <150% were classified into low liver regeneration group. Serial changes of laboratory data in both the groups are shown in Figure 3. Prothrombin time-INR on POD 3 and 5, serum bilirubin levels on POD 3 and 7, and serum aspartate aminotransferase levels on POD 3 and 5 were significantly higher in high liver regeneration group. On the other hand, platelet counts on POD 5 and serum *C*-reactive protein levels on POD 1 were lower in the high liver regeneration group.

### 2.3. Postoperative Changes of Serum Growth Factor Levels and Liver Regeneration

**Figure 4:**
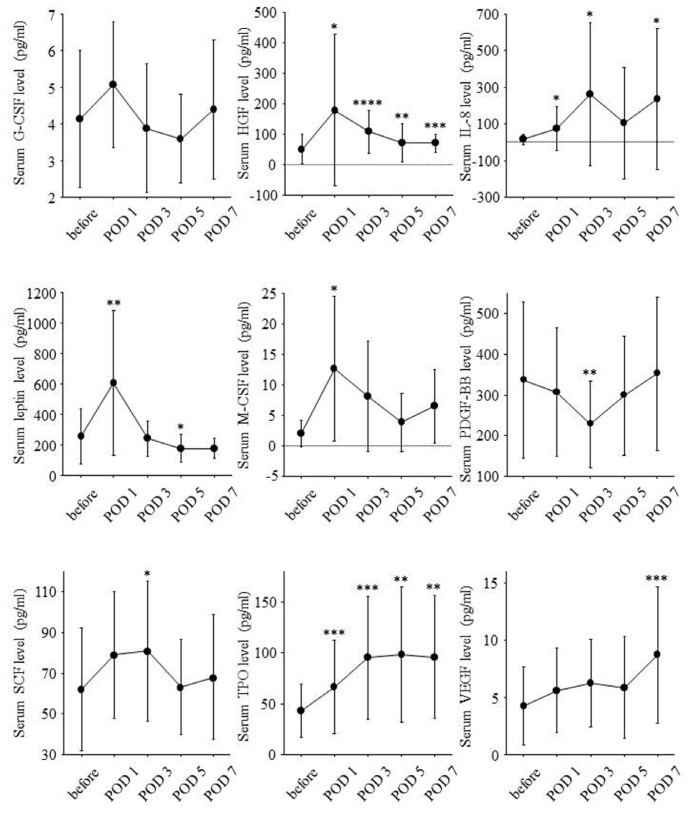
Serial changes of serum levels of nine growth factors during the clinical course. Serum levels of each growth factor before hepatectomy and on POD 1, 3, 5 and 7 were expressed as mean ± standard deviation. Before: before partial hepatectomy; POD: postoperative day; *: *p* < 0.05; **: *p* < 0.01; ***: *p* < 0.001; ****: *p* < 0.0001.

Serial changes of serum growth factor levels are shown in Figure 4. Postoperative changes in serum levels of HGF and leptin paralleled those in prothrombin time-INR and serum levels of bilirubin. The changes in serum levels of macrophage colony-stimulating factor (M-CSF) paralleled those in white blood cell counts. The changes in serum platelet-derived growth factor (PDGF)-BB levels paralleled those in platelet counts.

Liver resection rate was significantly correlated with serum M-CSF levels on POD 5 (*r* = 0.78, *p* = 0.037) and POD 7 (*r* = 0.81, *p* = 0.003) but not with serum HGF and leptin levels on POD 1. Remnant liver volume per body weight on POD 0 was inversely correlated with serum M-CSF levels on POD 5 (*r* = −0.76, *p* = 0.045) and POD 7 (*r* = −0.75, *p* = 0.010) and tended to be inversely correlated with serum HGF levels on POD 1 (*r* = −0.46, *p* = 0.076) and serum leptin levels on POD 1 (*r* = −0.47, *p* = 0.064).

**Figure 5:**
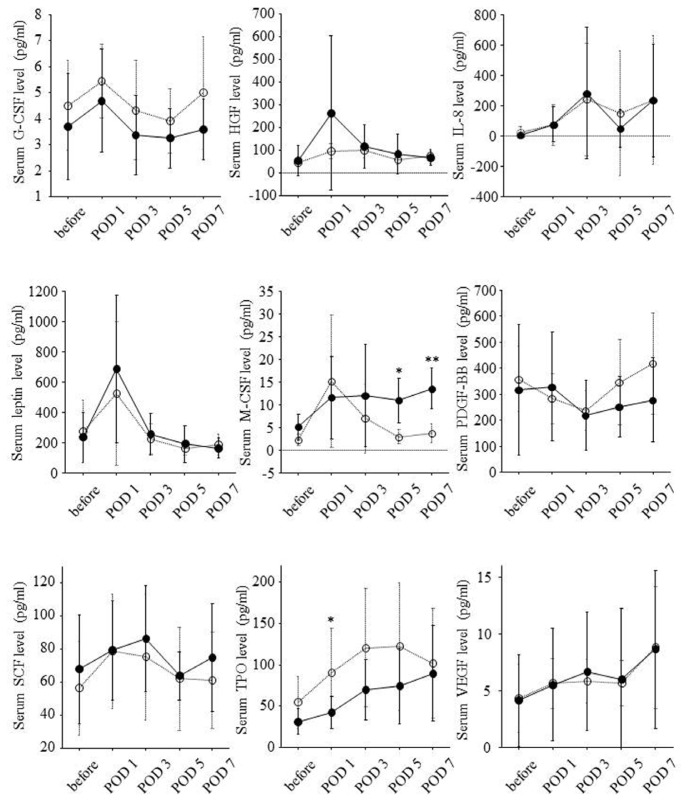
Associations of remnant liver volume per body weight on POD 0 with serial changes of serum levels of nine growth factors during the clinical course. Solid and dotted lines show serial changes of serum levels of each growth factor in eight patients with remnant liver volume per body weight on POD 0 of 10 cm^3^/kg or less and the other eight patients with remnant liver volume per body weight on POD 0 >10 cm^3^/kg, respectively. Serum levels of each growth factor before hepatectomy and on POD 1, 3, 5 and 7 were expressed as mean ± standard deviation. Before: before partial hepatectomy; POD: postoperative day; *: *p* < 0.05; **: *p* < 0.01.

According to remnant liver volume per body weight on POD 0, serial changes of serum growth factor levels are shown in Figure 5. In eight patients with remnant liver volume per body weight on POD 0 of 10 cm^3^/kg or less, serum M-CSF levels on POD 5 and POD 7 were significantly higher. On the other hand, serum TPO levels on POD 1 were lower in this group.

Ratio of liver volume on POD 14 to liver volume on POD 0 was significantly correlated with serum HGF levels on POD 1 (*r* = 0.54, *p* = 0.030), serum leptin levels on POD 1 (*r* = 0.54, *p* = 0.028), and serum M-CSF levels on POD 5 (*r* = 0.76, *p* = 0.047) and POD 7 (*r* = 0.80, *p* = 0.003). On the other hand, ratio of liver volume on POD 14 to liver volume on POD 0 was inversely correlated with serum PDGF-BB levels on POD 5 (*r* = −0.61, *p* = 0.011), and serum TPO levels on POD 1 (*r* = −0.60, *p* = 0.012).

**Figure 6:**
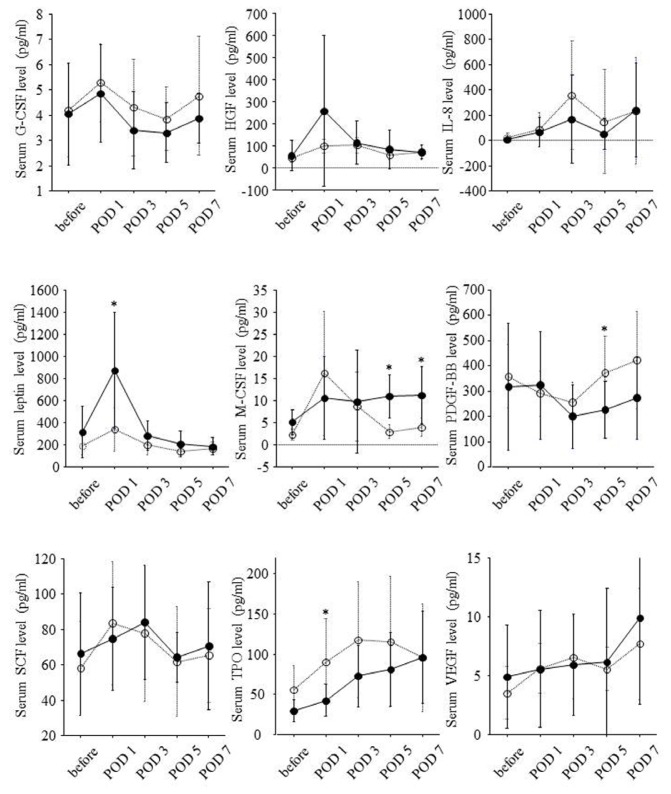
Associations of liver regeneration with serial changes of serum levels of nine growth factors during the clinical course. Solid and dotted lines show serial changes of serum levels of each growth factor in eight patients showing ratio of liver volume on POD 14 to liver volume on POD 0 of 150% or higher and the other eight patients showing this ratio <150%, respectively. Serum levels of each growth factor before hepatectomy and on POD 1, 3, 5 and 7 were expressed as mean ± standard deviation. Before: before partial hepatectomy; POD: postoperative day; *: *p* < 0.05.

Serial changes of serum growth factor levels in high liver regeneration group and low liver regeneration group are shown in Figure 6. Serum leptin levels on POD 1 and serum M-CSF levels on POD 5 and POD 7 were significantly higher in high liver regeneration group. Serum HGF levels on POD 1 seemed to be higher in high liver regeneration group although the difference was not significant. On the other hand, serum PDGF-BB levels on POD 5 and serum TPO levels on POD 1 were lower in the high liver regeneration group.

## 3. Discussion

The liver has strong potential to regenerate. Liver regeneration involves a complex interaction of the proliferation of resident hepatocytes and hepatocyte progenitor cells, the facilitation of angiogenesis, and the differentiation of hematopoietic stem cells into hepatocyte. However, the mechanism of liver regeneration in healthy humans has not been revealed yet. This study indicated that, after partial hepatectomy of the grade not exerting danger on a life, the smaller the remnant liver volume, the higher was liver regeneration, and that various growth factors intricately took parts in liver regeneration after partial hepatectomy. In particular, early-phase elevations of serum levels of HGF, leptin and M-CSF seemed to be associated with the acceleration of liver regeneration after partial hepatectomy.

As is well known, HGF is a potent factor for proliferation of hepatocyte. In this study, serum HGF levels on POD 1 were correlated with ratio of liver volume on POD 14 to liver volume on POD 0. These findings are consistent with the previous reports [6,7]. Recently, a clinical trial using recombinant HGF for acute liver failure has been reported, and it has been shown that intravenous administration of recombinant HGF is well-tolerated [11]. Further clinical trials are required to determine the effect of recombinant HGF on liver regeneration in humans.

Some studies have showed the relation of leptin with liver regeneration in animal models. In leptin-deficient ob/ob mice after toxic liver injury or partial hepatectomy, liver regeneration is impaired with down-regulated hepatic expression of TNF-α and IL-6, and leptin supplementation improves liver regeneration with up-regulated hepatic expression of TNF-α and IL-6 [12,13]. On the other hand, leptin does not directly up-regulate hepatocyte proliferation [14]. Leptin may accelerate liver regeneration through the release of cytokines such as TNF-α and IL-6 from non-parenchymal cells.

M-CSF is produced by non-parenchymal and parenchymal liver cells. In M-CSF-deficient mice, hepatic expressions of TNF-α and IL-6 are reduced, and proliferation of hepatocytes is impaired [15]. On the other hand, in M-CSF-deficient mice, M-CSF supplementation improves liver regeneration [15]. In addition, hepatocyte-like cells are reported to differentiate from peripheral blood monocytes under the stimulation of M-CSF [16]. M-CSF may take a part in liver regeneration through the proliferation of hepatocytes and the differentiation of hematopoietic stem cells into hepatocytes.

An appropriate intra-hepatic inflammatory response to liver injury has been shown to promote liver regeneration [17,18]. In this study, white blood cell counts on POD 1 were correlated with ratio of liver volume on POD 14 to liver volume on POD 0. However, serum *C*-reactive protein levels on POD 1 were shown to be inversely correlated with ratio of liver volume on POD 14 to liver volume on POD 0. This may be partially due to the interaction of *C*-reactive protein with leptin. *C*-reactive protein is reported to inhibit the binding of leptin to its receptor and attenuate its physiological functions [19]. In addition, *C*-reactive protein are shown to induce hepatic insulin-resistance which leads to poor liver regeneration [20,21].

Serum TPO levels in this study were gradually increased after partial hepatectomy, and these changes are consistent with the previous report [10]. TPO promotes liver regeneration after partial hepatectomy [5]. However, in this study, serum TPO levels on POD 1 were correlated with remnant liver volumes on POD 0. TPO is mainly produced by hepatocyte in response to thrombocytopenia when circulating platelet counts is decreased [22]. In this study, platelet counts abruptly decreased after the operation. In response to thrombocytopenia, serum TPO levels after the operation may be elevated in proportion to remnant liver volumes.

## 4. Materials and Methods

This study was approved by the Institutional Review Board at Okayama University Graduate School of Medicine, Dentistry, and Pharmaceutical Sciences, Okayama, Japan. Each patient was informed of the nature of the study and signed an informed consent form.

### 4.1. Study Population

Sixteen healthy liver donors who underwent partial hepatectomy between January 2000 and November 2010 were prospectively included in this study. Eight donors underwent a right lobectomy, three did an extended left lobectomy, two did a left lateral segmentectomy, one did a left lobectomy, and two did a right posterior segmentectomy, respectively.

### 4.2. Measurement of Serum Growth Factor Level

Sera were collected prior to the operation and on POD 1, 3, 5 and 7. Samples were frozen and stored at −80 °C until analysis.

Serum levels of the following growth factors were measured using the Bio-Plex Protein Array System (Bio-Rad Laboratories, Hercules, CA, USA): granulocyte colony-stimulating factor, HGF, IL-8, leptin, M-CSF, PDGF-BB, stem cell factor, and VEGF. In brief, the Bio-Plex Pro Standard and samples diluted in Serum Diluent were added to a 96-well filter plate and incubated with the antibody-coupled beads for 1 h with continuous shaking. The beads were washed three times with wash buffer to remove unbound protein and incubated with biotinylated detection antibodies for 30 min with continuous shaking. Following three washes, premixed streptavidin-phycoerythrin was added to each well and incubated for 30 min. After incubation, the beads were washed and re-suspended in assay buffer. The reaction mixture was quantified using the Bio-Plex protein array reader. Each growth factor level was automatically calculated by Bio-Plex Manager software using the appropriate standard curve.

Serum TPO level was measured using an enzyme-linked immunosorbent assay kit according to the manufacturer’s instructions (Quantikine Human TPO Immunoassay, R&D Systems, Minneapolis, MN, USA). Microplates were coated with manufacturer-provided monoclonal antibodies against TPO, and following the enzyme reaction the plates were measured using a microplate manager (BIO-RAD Laboratories, Hercules, CA, USA) and the optical density was determined at 450 nm.

### 4.3. Volumetric Study of Liver

Liver volumes were measured by multi-detector computed tomography (Aquilion 64, Toshiba Medical Systems Corporation, Otowara, Japan) using workstation (Virtual Place Advance Plus, Aze, Tokyo, Japan).

The liver resection rate (%) was calculated as follows: resected liver graft volume (cm^3^)/liver volume before the operation (cm^3^) × 100%.

### 4.4. Statistical Analysis

SPSS statistical program (release 11.0.1 J, SPSS, Chicago, IL, USA) was used for the statistical analysis.

Dichotomous variables were compared by the chi-squared test. Continuous variables were expressed as mean ± standard deviation (SD). Student’s *t*-test was used to evaluate differences in the continuous variables between two groups. The Pearson’s correlation test was used to evaluate the consistency in the continuous variables between two groups. *p*-values < 0.05 were considered significant.

## 5. Conclusions

After partial hepatectomy of the grade not exerting danger on a life, the smaller the remnant liver volume, the higher the liver regeneration is. This study indicates that various growth factors are associated with liver regeneration after partial hepatectomy in healthy humans. In particular, early-phase elevation of serum levels of HGF, leptin and M-CSF may be associated with accelerated liver regeneration. HGF, leptin and M-CSF possibly become new therapeutic agents for promoting liver regeneration. In addition, serial changes of serum levels of these growth factors may be early predictors of liver regeneration after hepatectomy. In order to confirm these findings in healthy humans, further studies are required.

## Figures and Tables

**Table 1:** Clinical characteristics of 16 healthy liver donors on admission.

Clinical Characteristics	Value
Age (year)	36 ± 12
Gender, female (%)	12 (75)
Height (cm)	161 ± 6
Body weight (kg)	59 ± 11
Body mass index (kg/m^2^)	22.8 ± 4.2

**Laboratory Data**	**Value**

White blood cell count (/mm^3^)	5574 ± 890
Hemoglobin concentration (g/dL)	13.4 ± 1.8
Platelet count (×10^4^/mm^3^)	24.7 ± 3.9
Bilirubin (mg/dL)	0.9 ± 0.5
Albumin (g/dL)	4.5 ± 0.3
Prothrombin time-international normalized ratio (INR)	0.98 ± 0.07
Aspartate aminotransferase (IU/L)	18 ± 4
Alanine aminotransferase (IU/L)	15 ± 9
C-reactive protein (mg/dL)	0.05 ± 0.07

**Table 2:** Liver graft type and changes of liver volume before and after hepatectomy.

Graft Type	Value
Liver graft type (left graft) n (%)	6 (38)
Liver graft type (right graft) n (%)	10 (62)

**Liver volume Change**	**Value**

Liver volume before hepatectomy (cm^3^)	1213 ± 206
Liver resection rate (%)	49 ± 20
Remnant liver volume on POD 0 (cm^3^)	622 ± 262
Remnant liver volume per body weight on POD 0 (cm^3^/kg)	10.7 ± 4.6
Liver volume on POD 14 (cm^3^)	917 ± 158
Ratio of liver volume on POD 14 to liver volume on POD 0 (%)	167 ± 54
